# Large Language Model–Supported Identification of Intellectual Disabilities in Clinical Free-Text Summaries: Mixed Methods Study

**DOI:** 10.2196/72256

**Published:** 2025-09-18

**Authors:** Aleksandra Edwards, Antonio F Pardiñas, George Kirov, Elliott Rees, Jose Camacho-Collados

**Affiliations:** 1 School of Computer Science and Informatics, Cardiff University, Cathays, Cardiff, CF24 4AG, United Kingdom, 1 029 2087 4812; 2 School of Medicine, Centre for Neuropsychiatric Genetics and Genomics, Division of Psychological Medicine and Clinical Neurosciences, Cardiff University, Cardiff, United Kingdom

**Keywords:** large language models, zero-shot learning, intellectual disabilities, clinical notes, text classification, information extraction, genetic analysis

## Abstract

**Background:**

Free-text clinical data are unstructured and narrative in nature, providing a rich source of patient information, but extracting research-quality clinical phenotypes from these data remains a challenge. Manually reviewing and extracting clinical phenotypes from free-text patient notes is a time-consuming process and not suitable for large-scale datasets. On the other hand, automatically extracting clinical phenotypes can be challenging because medical researchers lack gold-standard annotated references and other purpose-built resources, including software. Recent large language models (LLMs) can understand natural language instructions, which help them adapt to different domains and tasks without the need for specific training data. This makes them suitable for clinical applications, though their use in this field is limited.

**Objective:**

We aimed to develop an LLM pipeline based on the few-shot learning framework that could extract clinical information from free-text clinical summaries. We assessed the performance of this pipeline for classifying individuals with confirmed or suspected comorbid intellectual disability (ID) from clinical summaries of patients with severe mental illness and performed genetic validation of the results by testing whether individuals with LLM-defined ID carried more genetic variants known to confer risk of ID when compared with individuals without LLM-defined ID.

**Methods:**

We developed novel approaches for performing classification, based on an intermediate information extraction (IE) step and human-in-the-loop techniques. We evaluated two models: Fine-Tuned Language Text-To-Text Transfer Transformer (Flan-T5) and Large Language Model Architecture (LLaMA). The dataset comprised 1144 free-text clinical summaries, of which 314 were manually annotated and used as a gold standard for evaluating automated methods. We also used published genetic data from 547 individuals to perform a genetic validation of the classification results; Firth’s penalized logistic regression framework was used to test whether individuals with LLM-defined ID carry significantly more de novo variants in known developmental disorder risk genes than individuals without LLM-defined ID.

**Results:**

The results demonstrate that a 2-stage approach, combining IE with manual validation, can effectively identify individuals with suspected IDs from free-text patient records, requiring only a single training example per classification label. The best-performing method based on the Flan-T5 model and incorporating the IE step achieved an *F*_1_-score of 0.867. Individuals classified as having ID by the best performing model were significantly enriched for de novo variants in known developmental disorder risk genes (odds ratio 29.1, 95% CI 7.36-107; *P*=2.1×10^−5^).

**Conclusions:**

LLMs and in-context learning techniques combined with human-in-the-loop approaches can be highly beneficial for extraction and categorization of information from free-text clinical data. In this proof-of-concept study, we show that LLMs can be used to identify individuals with a severe mental illness who also have suspected ID, which is a biologically and clinically meaningful subgroup of patients.

## Introduction

### Background

Text classification of clinical data is a challenging problem due to highly specialized terminology, diverse document structures, and the heavy reliance of most methods on annotated data [1,2]. Standard approaches to overcome these challenges involve the use of task-specific knowledge and rule-based methods [3-6], which make them inapplicable to a wider range of tasks. This is because such methods rely heavily on handcrafted features and domain-specific rules, which do not generalize well beyond the narrow set of conditions they were designed for. Language models are neural network architectures trained to understand and generate human language by learning statistical patterns in large text corpora. Among the most influential are masked language models such as BERT (Bidirectional Encoder Representations From Transformers) and RoBERTa (Robustly Optimized BERT Approach) [7-12], which are pretrained on general-domain text and then fine-tuned—that is, further trained on labeled data for a specific downstream task such as text classification. In the biomedical and clinical domains, specialized variants such as BioBERT (BERT for Biomedical Text Mining) [13] and Clinical Bidirectional Encoder Representations From Transformers (ClinicalBERT) [14] have been developed by continuing pretraining on domain-specific corpora. These models have demonstrated improved performance in tasks like clinical concept extraction and classification. However, despite their success, they still require large amounts of annotated training data to achieve strong performance [15], which is often a limiting factor in clinical settings where labeled data are scarce [12,16-18]. Therefore, methods should ideally work without training data in a zero- or few-shot framework. Recent advances in natural language processing (NLP) have introduced alternative methods using text generation or large language models (LLMs) like Large Language Model Architecture (LLaMA) [19], which perform unseen tasks via in-context learning (prompting) [11,20-23]. Prompting involves giving the model natural language instructions that describe the task [24]. In few-shot prompting, these instructions are accompanied by a few training examples [24]. Unlike fine-tuning, prompting does not modify model weights, making it less resource intensive. Research shows that prompting can match or exceed the performance of standard fine-tuning [9,25]. Further gains in zero-shot settings have been achieved by fine-tuning models on task instructions, as in Fine-Tuned Language Net Text-To-Text Transfer Transformer (Flan-T5) [26,27]. However, their application in the clinical domain remains limited.

In contrast to previous work, we explore a novel approach for performing classification for clinical data. We use an intermediate information extraction (IE) step and human-in-the-loop framework to maximize the performance of in-context learning techniques and LLMs for one-shot setting. As a real-world example, we applied this procedure to free-text clinical summaries in a cohort of patients with severe mental illness and classified individuals with suspected comorbid intellectual disability (ID). These free-text clinical summaries were previously created from discharge patient notes and clinical interviews for research purposes. This is a challenging task as these summaries have diverse structure and terminology and may contain information related to different types of disabilities that can be hard to distinguish without detailed reports of the person’s cognitive functioning (eg, “learning disability” vs “intellectual disability”) [28]. Further, the presence of ID can be described using diverse terminology and be implicitly referred to within the text by recording outcomes and scores from different types of clinical tests and assessments without explicitly mentioning any disability. To provide evidence that our LLM approach can identify a biologically meaningful subgroup of patients, we used genetic data that is available in a subset of patients with schizophrenia to test whether individuals classified by LLMs to have suspected comorbid ID carry significantly more rare variants in ID-associated genes than individuals without LLM-defined ID.

### Related Work

We discuss relevant work using LLMs for extracting and classifying information within clinical texts, as well as outline challenges and research gaps within the literature (see sections “Text Classification for Clinical Text” and “Human-in-the-Loop–Based Approaches”). Finally, we present a use case from psychiatric genetics, a discipline with close relationships to the broader fields of clinical genetics and rare disease research (section “Genetic Analysis”).

#### Text Classification for Clinical Text

The generalization capabilities of text-generation models in few-shot settings make them suitable for the clinical domain, which often lacks annotated data and is also associated with limited access to datasets and language resources [29,30]. Thus, recent research in the clinical NLP field has focused on leveraging and evaluating the performance of such models for various tasks, including classification [1,31-37]. Some papers focus on providing dataset resources to support easier evaluation and benchmarking of LLMs in zero- and few-shot settings [1,37], as well as instruction or domain-trained models based on LLaMA [32,34,36]. These works show promising results for some downstream tasks.

A similar work to ours by Lu et al [33] proposes a knowledge-enhanced prompt learning method for classification of diagnosis from clinical texts. The approach is based on extracting relevant knowledge to the given diagnosis from heterogeneous knowledge resources and integrating it into a prompt sequence along with the clinical note. The authors experiment with more traditional neural network approaches, as well as masked language models. In contrast to this work, we analyze recently developed LLMs such as Flan-T5 and LLaMA, and we focus on exploring in-context learning techniques. In similar research by Fabregat et al [31], a Bidirectional Long Short-Term Memory (Bi-LSTM) architecture is used to extract the presence of named disabilities (including IDs) from clinical notes. In our paper, we extract intellectual disabilities, where for some patients these are not mentioned explicitly but instead referred to within the text by using different terminology as well as the results of relevant tests and assessments.

Another work introduces Generative Pre-Trained Transformer for Biomedical Text Generation and Mining (BioGPT) [38], a language model pretrained on large-scale biomedical literature and evaluated on a range of tasks such as relation extraction, question answering (eg, PubMedQA), document classification, and text generation. While it outperforms BERT-based models on these benchmarks, it is built on the older GPT-2 architecture and was trained exclusively on biomedical research articles. This limits its applicability to clinical contexts, particularly for understanding the more informal language often found in patient note summaries. Moreover, BioGPT is relatively small, with only 1.5 billion parameters, making it less capable compared to more recent and larger models such as LLaMA [19] and Flan-T5 [26], which offer improved generalization, reasoning, and contextual understanding across diverse domains.

Despite these recent attempts in leveraging LLMs for the clinical domain, most of the work in NLP-related applications for the health care domain is still relying on the use of more data-consuming approaches for text classification and IE [3-6]. In contrast to previous work, we explore different approaches for performing classification for patient notes using an intermediate IE step and human-in-the-loop approach to maximize the performance of in-context learning techniques and LLMs for one-shot setting.

#### Human-in-the-Loop–Based Approaches

Incorporating expert knowledge and human validation within automated approaches can have high benefits in the health care domain, given the undesirable consequences of misclassification inherent to some tasks and the subsequent need for highly accurate models [39]. Despite this, research in developing such hybrid approaches is very limited, focusing mainly on incorporating domain knowledge within more traditional rule-based and dictionary-based approaches for IE [40,41]. For instance, the authors of [40] use a human-in-the-loop approach for constructing a lexicon for extracting medication names from clinical records. The authors of [41] use a human-based verification step for building an ontology for structuring radiology reports. In contrast, our work is the first attempt in incorporating human knowledge with state-of-the-art NLP models to develop more accurate text classification approaches for scenarios with no training data available, except for a few examples.

#### Genetic Validation of ID Identified by LLM

Schizophrenia is a severe and highly heritable psychiatric disorder [42]. Impaired cognition is a core symptom of schizophrenia that strongly predicts worse functional outcomes [43]. Studies have demonstrated that rare and common genetic variation contributes to variation in cognitive ability, or proxies of cognition such as educational attainment, in schizophrenia. For example, we recently showed that genes associated with early onset developmental disorders (including ID) are enriched for damaging rare variants in individuals with schizophrenia and suspected comorbid ID [44]. In our previous work, we classified patients with schizophrenia with suspected comorbid ID by manually curating free-text clinical summaries. In this study, we analyzed the same exome-sequencing data and clinical summaries used in [44] and tested whether individuals with schizophrenia and LLM-classified comorbid ID carry significantly more damaging rare variants in known ID-associated genes compared with individuals with schizophrenia who did not have LLM-classified ID. Demonstrating that individuals with LLM-classified ID are enriched for such mutations would provide evidence that our LLM approach can identify a biologically meaningful subgroup of patients with schizophrenia.

### Objectives of This Study

Our objectives were as follows. First, we aimed to develop a novel 2-step approach that does not require training data for classifying individual patients with suspected ID within psychiatric clinical summaries. Toward this end, we explored approaches that use text generation models coupled with prompting techniques to perform IE for identifying ID-related information from the summaries, after which we performed classification on the extracted text. Second, we aimed to compare the performance of approaches that involve human-in-the-loop techniques and fully automated approaches. Third, we aimed to perform a multifaceted evaluation of model performance and analyze the effect of prompt information type (eg, task definitions vs examples provided as part of the prompt) on the performance for IE and text classification tasks. Lastly, we aimed to perform a genetic validation of the results via an experiment testing whether individuals with LLM-defined ID carry more genetic variants known to confer risk of ID when compared with individuals without LLM-defined ID.

## Methods

### Data

#### Corpus of Clinical Free-Text Data

The corpus contained free-text clinical summaries of 1144 individuals with severe mental illness, including schizophrenia and bipolar disorder. The summaries for each person contained information related to symptoms, reactions to prescribed treatments and medications, as well as any other observations that can be clinically relevant, such as the comorbid presence of other illnesses and developmental conditions (including ID). The summaries were not written for the purpose of the current project but at the time of patient recruitment for general research purposes. The summaries contained information from discharge summaries and clinical interviews with psychiatrists. Some clinical summaries also included the Schedules for Clinical Assessment in Neuropsychiatry (SCAN) interview [45], which is a semistructured clinical interview used to assess and diagnose psychiatric disorders. In addition, our dataset was a strong representation of real-world free-text clinical summaries, which often present challenges such as inconsistent structure, lack of labeled training data, and the use of diverse terminology where diseases and diagnoses may not be explicitly mentioned. Since the dataset was not specifically collected for this study, it offers potential for a wide range of research applications.

#### Dataset Annotation

We annotated 314 patient notes by manually curating the free text and identifying evidence of ID (see Table 1 for an overview of the annotation dataset). In addition to these 314 patient summaries, the best performing automatic approaches were used to classify an additional 830 patients.

**Table 1.** Overview of the dataset.

Class	Number of tests	Average number of tokens
ID^a^	29	222
No ID^b^	285	185
Total	314	190

a ID: intellectual disability.

b No ID: no evidence of intellectual disability present in the patient note (or lack of evidence).

### Methodology

#### Pipeline

We explored three different approaches for performing classification. First, we performed classification on the entire dataset to allow comparison with standard approaches. Second, we performed IE for identifying ID-related information from the summaries, and then we performed classification on the extracted text. Third, we proposed the use of a human-in-the-loop approach as an alternative to a fully automated approach where we use the IE step to extract only the relevant information to the task in a more concise format, which can support experts in performing more efficient and less error-prone annotation of documents.

#### Prompting Techniques

We used three prompting techniques (see Table 2) for both IE and classification tasks: *basic*, *definitions,* and *definitions+examples*. This allowed us to identify what type of prompt information (ie, information provided as part of the instruction) is more beneficial for the model’s performance.

1. *Basic prompt*: In this prompt, we simply provided a question to the model without further information about the task.
2. *Definitions*-enhanced prompt: In this prompt, we provided some descriptions about the tasks, that is, definition about intellectual disabilities, along with the question. We provided the same definition for both IE and classification models.
3. *Definitions+examples* prompt: In this prompt, along with the definition, we provided one example per label—for the IE task, we provided one example of an output that contains ID-related features and one that does not. The examples were selected randomly from the dataset. For generating data for the test sequences, we used a sampling method.

The definition used as part of the prompts for performing classification and IE is given in Textbox 1. The definition was taken from the *International Statistical Classification of Diseases, Tenth Revision* (*ICD-10* [46]), which is a medical classification list by the World Health Organization.

**Table 2.** Different prompt learning methods for clinical data.

	Information extraction prompts	Classification prompts
Basic prompt^a^	What is the evidence of intellectual disability the patient displays from the given patient note? Patient Note: SZ, IQ of 65., paranoid elements...[Answer]	Does the patient display any evidence of intellectual disability from the given patient note? Patient Note: SZ, IQ of 65., paranoid elements...[Answer]
Definitions^b^	[DEF] What is the evidence of intellectual disability the patient displays from the given patient note? Patient Note: SZ, IQ of 65., paranoid elements...[Answer]	[DEF] Does the patient display any evidence of intellectual disability from the given patient note? Patient Note: SZ, IQ of 65., paranoid elements...[Answer]
Definitions +examples^c^	[DEF] What is the evidence of intellectual disability the patient displays from the given patient note? Patient Note: SZ, IQ of 65., paranoid elements...[IQ of 65.] Patient Note: Started hallucinating; Education: Lawyer...[No evidence.] Patient Note: SZ, premature birth, attended special school...[Answer.]	[DEF] Does the patient display any evidence of intellectual disability from the given patient note? Patient Note: SZ, IQ of 65., paranoid elements... [Yes] Patient Note: Started hallucinating; Education: Lawyer...[No] Patient Note: SZ, premature birth, attended special school...[Answer]

a The ”Basic prompt” method simply adds questions to the clinical summaries.

b The “Definitions” prompt incorporates medical knowledge, ie, definition of intellectual disability, within the prompt.

c The “Definitions+examples” method adds two annotated examples for both tasks, information extraction (IE) and classification. For example, in the IE task, we provide two patient summaries: one that contains an intellectual disability–related feature (eg, “IQ of 65”) and one that does not. The first supports the presence of the feature and is labeled with the correct answer in square brackets (eg, [“IQ of 65”]). The second lacks such a feature and is labeled as “no evidence.” Additionally, new patient summaries marked with “[Answer]” are examples of where the model is expected to generate a prediction.

Textbox 1. **Definition used in prompts.**

You are a health care assistant, and you have been asked to identify if the given patient has intellectual disability (ID) for the given patient note. Please use the information about intellectual disabilities given below to extract the right information.

Intellectual disability (ID), previously known as mental retardation, is a term that is used when an individual has below-average intelligence or mental ability. Intellectual disability (ID) can be identified within the first two years of a child’s life if he or she has more severe intellectual disabilities. However, mild intellectual disability may not be identifiable until the child reaches school-age, when challenges with academic learning become present. While it typically occurs during the developmental periods, it is also possible for intellectual disability to develop later as the result of illness or brain injury. Signs and symptoms of intellectual disabilities include: premature birth, delayed development, learning and developing more slowly than other children same age, difficulty communicating or socialising with others, lower than average scores on IQ tests, difficulties talking or talking late, having problems remembering things, inability to connect actions with consequences, difficulty with problem-solving or logical thinking, trouble learning in school, need to attend special school, inability to do everyday tasks like getting dressed or using the restroom without help.

### Experimental Setup

In this section, we describe our experimental setting for the task of identifying patients with ID in free-text clinical data.

#### Comparison Models

We performed analysis with LLaMA 2 [19] as a representative of a large autoregressive generation model with 70 billion parameters. As a representative of a smaller but instruction-tuned model, we used Flan-T5 [26], in particular its XXL version with 11 billion parameters. The model was fine-tuned using the Flan instruction tuning tasks collection [26]. The collection also included datasets related to the medical domain and classification tasks. We used the XXL version with 11 billion parameters. We downloaded the models from Hugging Face [47]. Due to the sensitivity of the patient notes, we decided against using OpenAI models or other external application programming interfaces requiring data upload for performing analysis. We chose LLaMA and Flan-T5 for our experiments because they are among the most recent, largest, and most versatile language models available, demonstrating strong performance across a wide range of tasks. Notably, Flan-T5’s training data includes medical content, which enhances its ability to understand and generate clinically relevant text, which makes it especially suitable for our use case. The model parameters we used for summarization and text classification are as follows: for Flan-T5, we used a temperature of 0.7 and a maximum of 10 and 30 generated tokens for classification and summarization, respectively. These are the default values recommended for these models. We used approximately 24 hours of GPU budget and the Nvidia RTX 4090 GPU (Nvidia Corporation). The implementation is available at GitHub [48].

#### Evaluation

We report classification results based on precision, recall, and standard micro- and macroaveraged *F*_1_ [49]. We judged the quality of the data generated during the IE stage based on the performance of the classification model applied to the IE output.

#### Genetic Analysis

We analyzed published genetic data that we previously generated from 547 individuals with schizophrenia who also have free-text clinical data (the paper by Rammos et al [44] provides a full description of this sample and genetic dataset). In this genetic analysis, we compared the rate of de novo variants (ie, newly arising mutations that were not inherited from either parent) in known developmental disorder risk genes between patients with suspected comorbid ID and patients without ID using Firth’s penalized likelihood logistic regression test, covarying for 10 principal components that were derived from the genetic data to control for genetic ancestry and sex. In the genetic analysis, we examined three classifications of ID: (1) ID defined through manual curation of the clinical summaries; (2) ID defined by the best performing fully automated NLP classification model; and (3) ID by the best performing human-in-the-loop classification model. Based on our previous study, we expected patients with suspected comorbid ID to have significantly more de novo variants in developmental disorder risk genes than patients without comorbid ID [44].

### Ethical Considerations

The following committees provided ethical approval for this study: Ethics Commission, Higher Medical University, Plovdiv, 4002 V Aprilov Blvd 15a; Protocol Ethics Committee to the Alexander University Hospital, Sofia 1431, 1 St G Sofiisk St, Local Ethics Committee, District Dispensary for psychiatric disorders, Russe, bul; Ethics Committee at the State Psychiatric Hospital “Dr Georgi Kisiov,” Radnevo, 6269, Magda Petkanova St 1, Radnevo (protocol of 2.10.2000); and Ethics Committee at the District Dispensary for psychiatric disorders, Blagoevgrad (protocol N2/2000). In the United Kingdom, the project was approved by the Bro Taf Local Research Ethics Committee, Churchill House, 17 Churchill Way, Cardiff CF10 2TW, protocol 02/4523. All study participants provided written informed consent with the ability to opt out at any time. Data were deidentified prior to analysis, and no identifying participant information is presented in this study. Participants were not compensated for their inclusion in the study.

The use of LLMs in processing clinical notes raises significant ethical challenges that must be carefully addressed. Preserving patient privacy is an essential priority when it comes to analyzing clinical notes. Even when deidentified, the risk of reidentification remains, especially with powerful models capable of memorizing or inferring sensitive data. Model transparency is another key concern, making it difficult to interpret LLM decision-making processes and validate their outputs in clinical settings where accountability is crucial. Further, bias in training data can propagate or even amplify existing disparities in health care, potentially leading to skewed predictions [50,51].

While a comprehensive treatment of these issues lies outside the scope of our current work, we actively considered these ethical concerns throughout our research. To mitigate risks, we have not released the dataset publicly, and we limited our experiments to open-source models. Moreover, our proposed integration of human-in-the-loop methods provides an additional layer of oversight, helping to ensure more responsible and secure use of LLMs in sensitive clinical applications.

## Results

### The Role of IE

**Figure 1.**
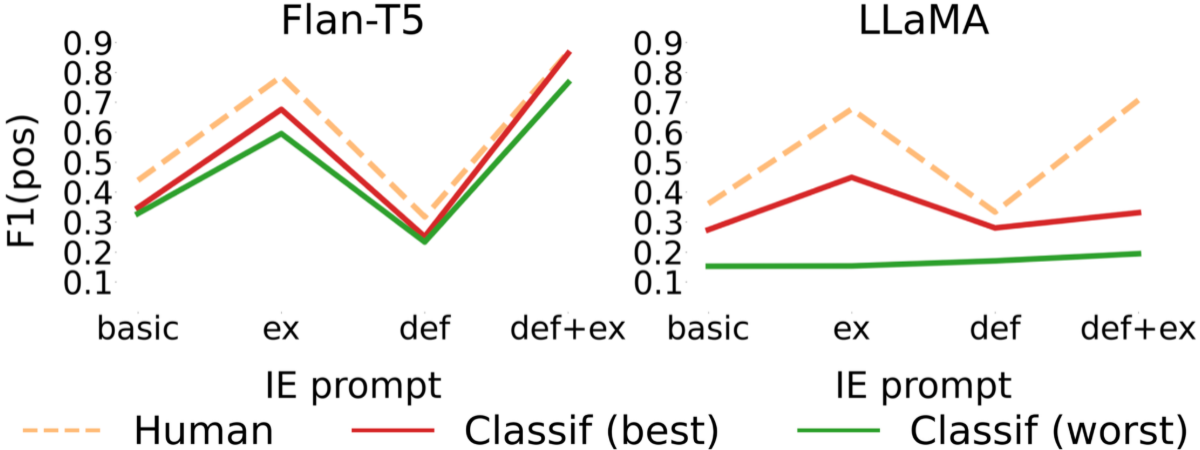
Comparison between classification approaches, that is, using entire notes versus using IE where “F1(pos)” refers to the *F*_1_-score for the positive class. “IE (avg),” “IE (best),” and “IE (worst)” refer to the average, best, and worst results, respectively, for the IE approach per classification prompt. Classif: classification; Flan-T5: Fine-Tuned Language Net Text-To-Text Transfer Transformer; IE: information extraction; LLaMA: Large Language Model Architecture.

Results in Table 3 show that Flan-T5 consistently outperforms LLaMA 2 regardless of classification approach or prompt use. This suggests that a smaller but instruction-tuned model, pretrained using datasets relevant to the clinical domain, is more suitable for classification in low-resource settings when compared with a bigger model. Further, results show that the performance of IE approaches is highly dependent on the prompt used, where the difference in *F*_1_-score for the positive class between the best and worst performing IE approach is around 0.5 (see Figure 1). A trend in the performance of both models (see Table 3) shows that a prompt combining a description of the task and examples leads to the best classification results versus a basic prompt or a prompt based only on definitions. Further, the best results were achieved with the Flan-T5 model using the IE intermediate step with a “definitions+examples” prompt informing both classification and IE (precision=1.00; recall=0.758) versus performing classification on the entire notes (*P*=.87; *r*=0.77).

These results show the potential of LLMs and in-context learning techniques to support classification tasks in the clinical domain. However, the performance of Flan-T5 varies with different prompts, whereas the LLaMA model achieves consistent improvements in classification regardless of the prompt. These findings highlight the prompt sensitivity issue in language models, particularly in smaller models like Flan-T5.

**Table 3.** Classification results.

Input and classification^a^		Flan-T5^b^					LLaMA^c^				
		Prec^d^	Rec^e^	F1(pos)^f^	Macro^g^	Acc^h^	Prec	Rec	F1(pos)	Macro	Acc
Full notes											
	basic	0.741	0.793	0.767	0.871	0.955	0.083	0.279	0.127	0.403	0.467
	examples	0.676	0.766	0.718	0.843	0.942	0.122	0.758	0.210	0.406	0.471
	def	0.741	0.689	0.714	0.843	0.948	0.113	0.379	0.174	0.482	0.665
	def+ex	0.869	0.689	0.769	0.874	0.961	0.125	0.689	0.212	0.435	0.522
IE (basic)											
	basic	0.205	0.827	0.328	0.562	0.686	0.103	0.295	0.152	0.423	0.505
	examples	0.217	0.896	0.348	0.572	0.690	0.153	0.750	0.254	0.447	0.515
	def	0.180	0.827	0.296	0.525	0.635	0.164	0.464	0.242	0.520	0.681
	def+ex	0.220	0.827	0.348	0.582	0.712	0.174	0.642	0.274	0.511	0.625
	human	0.294	0.862	0.439	0.657	0.796	0.277	0.517	0.361	0.618	0.792
IE (def)											
	basic	0.135	0.827	0.233	0.426	0.491	0.121	0.285	0.170	0.511	0.749
	examples	0.141	0.896	0.243	0.423	0.479	0.127	0.517	0.204	0.479	0.625
	def	0.126	0.862	0.221	0.388	0.434	0.046	0.178	0.074	0.408	0.598
	def+ex	0.146	0.896	0.251	0.438	0.501	0.184	0.586	0.280	0.554	0.721
	human	0.896	0.193	0.317	0.536	0.639	0.583	0.233	0.333	0.642	0.910
IE (ex)											
	basic	0.487	0.759	0.595	0.770	0.903	0.163	0.571	0.253	0.531	0.696
	examples	0.489	0.828	0.615	0.780	0.904	0.256	0.714	0.377	0.624	0.787
	def	0.500	0.828	0.623	0.785	0.907	0.093	0.428	0.153	0.434	0.574
	def+ex	0.589	0.793	0.676	0.818	0.929	0.327	0.714	0.449	0.678	0.841
	human	0.815	0.759	0.786	0.882	0.962	0.666	0.689	0.677	0.822	0.938
IE (def+ex)											
	basic	1.000	0.621	0.765	0.873	0.964	0.160	0.592	0.252	0.528	0.691
	examples	1.000	0.655	0.792	0.887	0.967	0.169	0.857	0.282	0.505	0.605
	def	1.000	0.655	0.792	0.887	0.967	0.117	0.571	0.194	0.450	0.569
	def+ex	1.000	0.758	0.863	0.887	0.968	0.205	0.857	0.331	0.562	0.686
	human	1.000	0.767	0.867	0.928	0.978	0.793	0.639	0.707	0.836	0.939

a The “input” refers to the type of input passed to the classifier, which is either an entire note or the output from the information extraction (IE) step where “IE (basic),” “IE (def),” “IE (ex),” and “IE (def+ex)” show prompt types used for the IE step. The “prompt (class)” refers to the prompt types we used for classification, that is, “basic,” “examples,” “def,” and “def+ex.” The “human” classification refers to the human-in-the-loop classification approach.

b Flan-T5: Fine-Tuned Language Net Text-To-Text Transfer Transformer.

c LLaMA: Large Language Model Architecture.

d Prec: precision.

e Rec: recall.

f F1(pos): *F*_1_ for the positive class.

g macro: *F*_1_-macro.

h Acc: accuracy score.

### Human-in-the-Loop Approach

**Figure 2.**
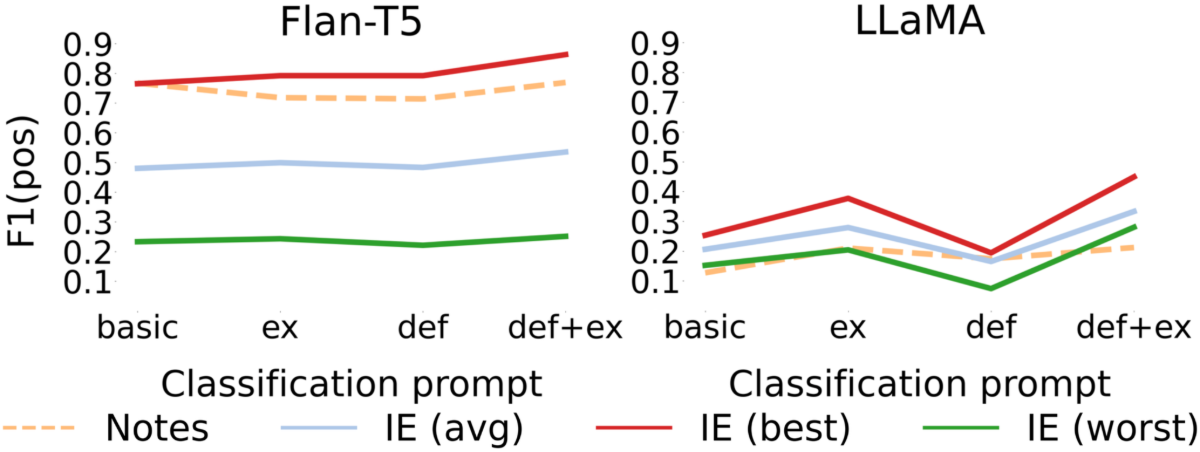
Comparison between fully automated IE-based classification and human-in-the-loop approach where “F1(pos)” refers to the *F*_1_-score for the positive class, “classif (best)” and “classif (worst)” refer to the best and worst classification results, respectively, per given IE prompt. Flan-T5: Fine-Tuned Language Net Text-To-Text Transfer Transformer; IE: information extraction; LLaMA: Large Language Model Architecture.

Results in Figure 2 and Table 3 show that combining IE for extracting task-relevant information and manual classification can support more accurate and less time-consuming classification versus using fully manual or fully automated methods. The human-in-the-loop approach led to better classification performance, especially for the LLaMA model where the difference in F1(pos) between the best performing IE approach and human-based method is 0.436. For the Flan-T5 model, the improvement in F1(pos) is 0.003 (F1(pos)=0.867 vs F1(pos)=0.863). Further, the average length of extracted passages using the FLan-T5 model is 3 tokens, whereas the average length for the entire note is 190 tokens. This shows that the IE step combined with the human-in-the-loop approach can be beneficial for supporting verification or the conduct of more efficient expert annotations. This could be a good alternative to a fully automated classification, especially in the health care domain where accuracy of models and reliability of results are of high importance.

### Genetic Validation of Different Classification Approaches

In all three classifications of ID (manual curation of clinical summaries, best performing fully automated NLP model, and performing human-in-the-loop model; see Table 3), damaging de novo variants were significantly enriched in patients with schizophrenia with suspected comorbid ID compared with patients with schizophrenia without ID (Table 4). De novo variants were most strongly enriched in the schizophrenia ID group defined by the human-in-the-loop classification (odds ratio 29.1, 95% CI 7.36-107), with the weakest enrichment observed in the fully automated classification (odds ratio 15.7, 95% CI 3.58-57.5). The same set of de novo variants was observed in the ID and non-ID patient groups in the human-in-the-loop and manual curation classifications, but a greater enrichment was observed in the human-in-the-loop classification test as fewer individuals were classified to have ID (14 in the human-in-the-loop classification vs 18 in the manual curation classification; Table 4).

To investigate why fewer people were found to have ID in the human-in-the-loop classification dataset, we examined the overlap of individuals with ID in this dataset and the manually curated classification dataset. We also reexamined the clinical information for individuals found to have ID in only one dataset. Eleven individuals with schizophrenia were recorded as having ID in both the manually curated and human-in-the-loop classification datasets. Among the 7 individuals who were found to have ID only in the manually curated classification dataset, 2 had clear evidence of having ID, 2 had ambiguous evidence of having ID, and 3 had no evidence of having ID. Among the 3 individuals found to have ID only in the human-in-the-loop classification dataset, 1 had clear evidence of having ID and 2 had ambiguous evidence of having ID. These results provide suggestive evidence that the human-in-the-loop approach produces fewer false positive ID classifications when compared with the manually curated ID set.

**Table 4.** Enrichment of de novo variants in individuals with SZ^a^ and comorbid ID^b^.

Classifier^c^	Patients with SZ and ID, n	Patients with SZ but without ID, n	*P* value	Odds ratio (95% CI)	Variants in ID group, n (rate)	Variants in no-ID group, n (rate)
Manual curation	18	529	7.1×10^–5^	21.1 (5.48-74.0)	4 (0.22)	7 (0.013)
Automatic model	16	531	9.3×10^–5^	15.7 (3.58-57.5)	3 (0.19)	8 (0.015)
Human-in-the-loop	14	533	2.1×10^–5^	29.1 (7.36-107)	4 (0.29)	7 (0.013)

a SZ: schizophrenia.

b ID: intellectual disability.

c The numbers of patients with ID and without ID are presented across three ID classifications where “manual curation” refers to manual annotations performed by a domain expert, “automatic model” refers to the best performing fully automatic model in Table 3, and “human-in-the-loop” refers to the best performing human-in-the-loop approach in Table 3.

## Discussion

### Principal Findings

Our findings show the potential of LLMs to facilitate different tasks in the clinical domain, such as classification and IE when only a few training examples are available. We compared three approaches for classifying ID from the clinical summaries of individuals with severe mental illness and found that using an IE step as part of a classification pipeline based on the Flan-T5 model and informed by a prompt combining definitions and examples achieved a precision of 1.00 and *F*_1_ of 0.867 for the positive class. Further improvements to classification were found when using the human-in-the-loop approach, a process where a human must review short NLP-derived summaries instead of the full clinical dataset. These findings open interesting research avenues in building hybrid approaches, which combine the benefits LLMs offer for extracting relevant information in a fast and efficient manner with the knowledge of experts. These kinds of methods can be suitable for classification tasks which are considered challenging even for domain experts, as well as for sensitive tasks that require high accuracy, which is typically hard to achieve when labeled training data is scarce. Moreover, the ability of our methods to accurately classify ID was supported by our genetic analysis, which found the NLP-defined subgroup of people with schizophrenia and ID to be enriched for genetic variants in genes known to be associated with ID. From conversations with geneticists in the area, we found that manual annotation of comorbidities is often based on keyword search, since reading of thousands of clinical notes is typically unfeasible. Our findings therefore suggest that NLP-based approaches can be used to validate or improve classification annotations derived from the manual curation of free-text clinical summaries, which is prone to human error. Further work is required to test these findings in routinely collected health care data, such as that captured in electronic health records, and how to best integrate into genetic analysis research and beyond the detection of IDs which was used as a first approximation to the more general problem of extracting information from free-text clinical summaries.

### Importance of Both Automation and Human Intervention in Health Care Applications

We have performed further analysis comparing the execution time efficiency of the best performing automatic and human-in-the-loop approaches to the manual methods used by experts for annotating the data (see Table 5). Specifically, the manual curation involved careful reading through the entire dataset to classify them. In the keyword search, experts perform simple searches using the operating system's search functionality to find potential class candidates. For these experiments, they used the keywords “IQ” and “mental handicap”. The results in Table 5 show the benefits of the automatic and human-in-the-loop approach versus the human-based annotation where the fully automated method is more than 20 times faster than manual curation and keyword-based search. In addition, keyword search has a slightly lower *F*_1_-score than automatic approaches. The reason for this is the limited number of keywords used in experiments. However, these results still highlight potential problems with such an approach where a careful selection of keywords is needed as well as a good knowledge of the corpus. Further, the human-in-the-loop approach performs at a very similar execution time to the fully automated approach but produces a higher *F*_1_-score. Perhaps even more importantly, the fact that an expert can be involved in the process provides increased reassurance compared to a fully automatic process. This shows that semiautomatic approaches can be an efficient and more reliable option versus full automation for the health care domain where high accuracy of models is required. However, research in this field is still very limited. Our work is one of the first attempts to incorporate manual verification and LLMs to create more reliable and less data-consuming approaches for classification of clinical free-text data.

**Table 5.** Comparison of the performance of classification approaches in terms of time taken for annotating 314 clinical summaries.

Approach	Time	F1(pos)^a^
Manual curation^b^	∼10 h	1.000
Keyword search^c^	∼2 h	0.845
Automatic model^d^	∼5 min	0.863
Human-in-the-loop^e^	∼20 min	0.863

a F1(pos): *F*_1_ for the positive class.

b “Manual curation” refers to manual annotations performed by a domain expert.

c “Keyword search” refers to CTRL-F–based search using the keywords “IQ” and “mental handicap.”

d “Automatic model” refers to the best performing fully automatic model in Table 3.

e “Human-in-the-loop” refers to the best performing human-in-the-loop approach in Table 3.

### Limitations

First, this study is based on a single free-text clinical dataset containing a summary of real-world clinical data, which was developed for research purposes. This dataset is not a direct copy of routinely collected clinical data, which may limit the generalization of the results. To mitigate this issue, we used unsupervised approaches that are aimed at modeling the problem at hand, without relying on training data that may easily be overfit to our own data. Nonetheless, these experiments would ideally need to be replicated on similar cohorts to better understand the strengths and limitations. Second, and related to the first point, the experiments were performed in English only, which is the language of the corpus of clinical summaries. Third, for this analysis, we simplified the task into a binary problem, in which patients have suspected comorbid ID or no evidence of ID. However, the clinical course of many medical conditions is complex, with high heterogeneity in the type and severity of symptoms both across individuals and within individuals over time. This is particularly true for mental health conditions, where individuals often receive different diagnoses during their life. It is therefore important for future studies to consider how models can adapt and capture these complex clinical phenotypes from longitudinal health care data. Although outside the scope of this paper, we note that our approach offers a promising path for this task, since it leverages in-context learning and integrates both labeled data and regularly updated, domain-specific external resources.

Another limitation of our study was that all manual annotations were performed by a single expert. This was due to the complexity and domain-specific expertise required for the task, making it unfeasible to involve multiple annotators. To ensure the reliability of the automated methods, we validated them not only against human annotations but also by using genetic data to confirm that individuals with NLP-defined ID are enriched for genetic variants known to be associated with ID.

### Conclusions

In this study, we analyze how language models such as Flan-T5 and LLaMA 2 combined with in-context learning can be utilized for classifying individuals with severe mental illness and suspected ID from free-text clinical summaries. We propose the use of an intermediate IE step for extracting relevant parts of the notes before classification. Our results show that such techniques can help improve the performance of LLMs in one-shot settings when combined with a prompt that provides both information about the task and relevant examples. In addition, we propose a human-in-the-loop approach as an alternative to fully automated classification, where the IE step is used to extract succinct parts of the notes related to the task, which can be used to support faster and less error-prone manual classification. Approaches based on this pipeline and the Flan-T5 model showed promising results and were validated in a proof-of-concept genetic analysis, which found individuals classified by NLP to have ID were enriched for genetic variants known to contribute to developmental disorders.

## Abbreviations

BERT: Bidirectional Encoder Representations From Transformers
Bi-LSTM: Bidirectional Long Short-Term Memory
BioBERT: Bidirectional Encoder Representations From Transformers for Biomedical Text Mining
BioGPT: Generative Pre-Trained Transformer for Biomedical Text Generation and Mining
ClinicalBERT: Clinical Bidirectional Encoder Representations From Transformers
Flan-T5: Fine-Tuned Language Net Text-To-Text Transfer Transformer
*ICD-10*: *International Statistical Classification of Diseases, Tenth Revision*
ID: intellectual disability
IE: information extraction
LLaMA: Large Language Model Architecture
LLM: large language model
NLP: natural language processing
RoBERTa: Robustly Optimized BERT Approach
SCAN: Schedules for Clinical Assessment in Neuropsychiatry
